# Changes in H-Reflex Recruitment After Trans-Spinal Direct Current Stimulation With Multiple Electrode Configurations

**DOI:** 10.3389/fnins.2018.00151

**Published:** 2018-03-28

**Authors:** Alexander Kuck, Dick F. Stegeman, Herman van der Kooij, Edwin H. F. van Asseldonk

**Affiliations:** ^1^Laboratory of Biomechanical Engineering, Department of Engineering Technology, University of Twente, Enschede, Netherlands; ^2^Neuronal Rhythms in Movement Unit, Okinawa Institute of Science and Technology Graduate University, Onna-son, Japan; ^3^Department of Neurology/Clinical Neurophysiology, Radboud University Medical Center, Donders Institute for Brain, Cognition and Behavior, Nijmegen, Netherlands; ^4^Department of Biomechanical Engineering, Faculty of Mechanical, Maritime and Materials Engineering, Delft University of Technology, Delft, Netherlands

**Keywords:** tsDCS, H-reflex, neuromodulation, spinal cord, neurorehabilitation

## Abstract

Trans-spinal direct current stimulation (tsDCS) is an electro-modulatory tool with possible application in the rehabilitation of spinal cord injury. TsDCS generates a small electric field, aiming to induce lasting, functional neuromodulation in the targeted neuronal networks. Earlier studies have shown significant modulatory effects after application of lumbar tsDCS. However, for clinical application, a better understanding of application specific factors is required. Our goal was to investigate the effect of different electrode configurations using lumbar spinal tsDCS on spinal excitability. We applied tsDCS (2.5 mA, 15 min) in 10 healthy subjects with three different electrode configurations: (1) Anode and cathode placed over vertebra T11, and the posterior left shoulder respectively (LSC-S) (one polarity), and (2) Both electrodes placed in equal distance (ED) (7 cm) above and below vertebra T11, investigated for two polarities (ED-Anodal/Cathodal). The soleus H-Reflex is measured before, during and after tsDCS in either electrode configuration or a sham condition. To account for genetic predispositions in response to direct current stimulation, subject BDNF genotype was assessed. Stimulation in configuration ED-Cathodal induced an amplitude reduction of the H-reflex, 30 min after tsDCS with respect to baseline, whereas none of the other configurations led to significant post intervention effects. BDNF genotype did not correlate with post intervention effects. Furthermore, we failed to replicate effects shown by a previous study, which highlights the need for a better understanding of methodological and subject specific influences on tsDCS outcome. The H-reflex depression after tsDCS (Config. ED-Cathodal) provides new insights and may foster our understanding of the working mechanism of tsDCS.

## Introduction

The targeted application of electrotherapy to the rehabilitation of nervous system disorders has been a lasting vision in rehabilitation research. In recent years, trans-spinal direct current stimulation (tsDCS), a variant of transcranial Direct Current Stimulation (tDCS), has received an increasing scientific interest as a proposed novel electrotherapeutic protocol. Aiming to modulate pathways in the Spinal Cord, tsDCS imposes a small electric field (EF) to the spinal neural circuitry. The ultimate goal is the ability to facilitate spinal plasticity and promote rehabilitation after neural injury of the spinal cord, via a meaningful and targeted application of tsDCS, in combination with established rehabilitation techniques.

Earlier research on the neural effects of DC stimulation, which originates mainly from studies on direct current stimulation of the cortex, has revealed a collection of multiple neural working mechanisms (Bikson et al., 2013; Miranda, 2013; Ruffini et al., 2013) depending on electric field magnitude and direction (Salvador et al., 2010; Dmochowski et al., 2011; Rampersad et al., 2014), the underlying neuroanatomy and its alignment with the imposed EF (Tranchina and Nicholson, 1986; Radman et al., 2009; Arlotti et al., 2012; Kabakov et al., 2012) as well as the ongoing neural activity (Reato et al., 2010; Ranieri et al., 2012; Bikson et al., 2013; Lapenta et al., 2013) and genetic predispositions (Bikson et al., 2013; Lamy and Boakye, 2013; Chhabra et al., 2015).

Consequently, previous studies which have applied tsDC-stimulation on the lumbar spinal cord, also revealed a complex picture of its effects on the spinal motor circuitry (for a thorough overview, see: Cogiamanian et al., 2012). It has been shown, that anodal tsDCS can lead to a significant increase (Hubli et al., 2013), or more specifically, a left shift of the H-reflex recruitment curve (Lamy et al., 2012), whereas cathodal stimulation had no significant effect. Also, cathodal and anodal tsDCS, were able to up- and downregulate cortically evoked motor evoked potentials (MEPs) at lumbar spinal level respectively (Bocci et al., 2015). Furthermore, it was shown that lumbar tsDCS has a significant modulatory effect on spinal reflex presynaptic inhibition (Yamaguchi et al., 2013) and post-activation depression (Winkler et al., 2010). As for tDCS, also in tsDCS genetic factors have been implicated to have an effect on the outcome of DC stimulation protocols (Chhabra et al., 2015). In particular, a polymorphism (*Val66Met*) of Brain-derived Neurotropic Factor (*BDNF*), has been of particular interest. Thereby, Lamy and Boakye showed that the H-reflex recruitment curve modulation after tsDCS significantly differs in carriers and non-carriers of the *BDNF Met allele* (Lamy and Boakye, 2013).

For a successful application of tsDCS in a clinical setting, a better understanding of its application specific effects is needed. This includes knowledge about proper electrode placement, the resulting electric field at the target region and its effects on the targeted neural circuitry. Based on studies simulating the electric field generated by transcutaneous DC stimulation, the EF-vector for a pair of surface electrodes is expected to be largest and tangential to the skin—surface about half-way between electrodes. Below the electrodes the EF vector will be comparably lower and perpendicular to the skin-surface (Kuck et al., 2017). Given that the neural effect of DC stimulation is dependent on EF strength and direction, the modulatory outcomes are expected to vary across tsDCS protocols employing different electrode configurations. However, since all previous studies utilized a similar electrode configuration (passive electrode on the shoulder, active electrode above the lumbar spinal cord), current knowledge does not allow conclusions about electrode placement specific effects of tsDCS.

**Figure 1:**
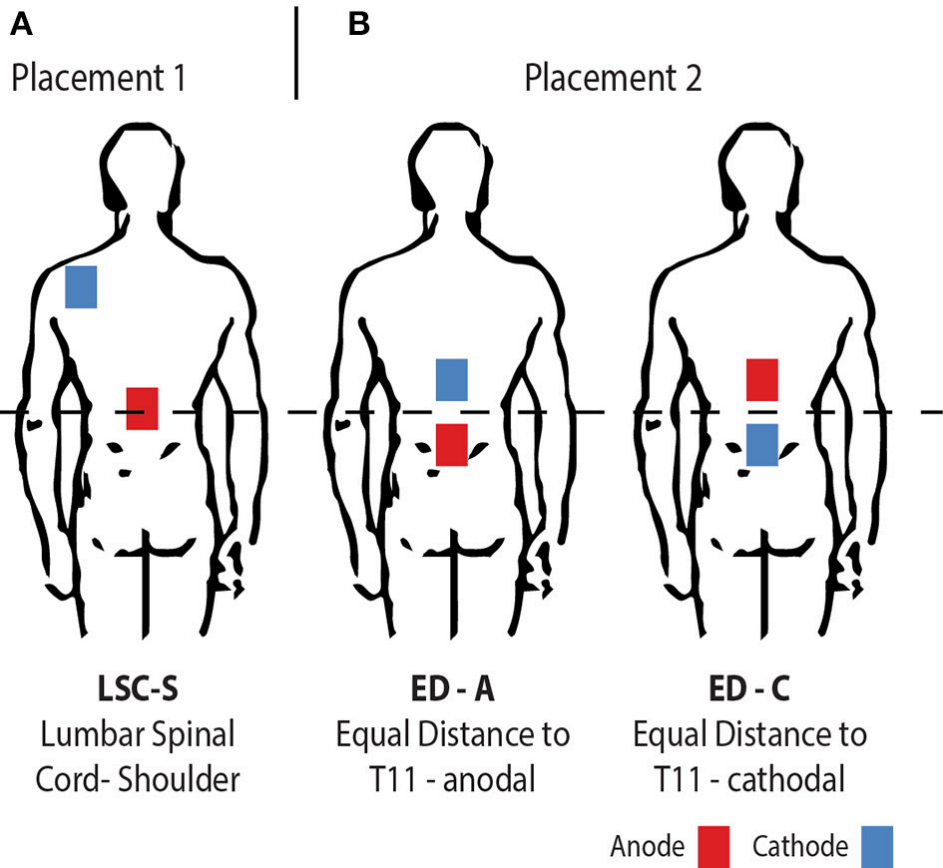
Illustration of the electrode placement configurations investigated. From left to right: **(A)** The traditionally used placement with one anodal electrode centered on the lumbar spinal cord and a return electrode placed on the left posterior shoulder (LSC-S); **(B)** Both electrodes placed in equal distance to the lumbar spinal cord, for opposite polarities (ED-A and ED-C).

In this study, our goal was therefore to investigate the effect of tsDCS on the soleus H-reflex with three electrode configurations (Figure 1). We compared the commonly used electrode configuration to a new bipolar electrode placement, with both electrodes placed in equal distance, above and below the lumbar spinal cord. We measured the soleus H-Reflex before, during and after tsDCS with both configurations and a sham condition. The commonly used placement was tested in anodal configuration only, which had previously shown to be effective in modulating the soleus H-Reflex (Lamy et al., 2012; Hubli et al., 2013). To probe for polarity specific effects, for the new electrode placement both anodal and cathodal configuration were investigated. We were primarily interested in the changes in H-Reflex amplitude post-tsDCS with respect to baseline. Additionally, we tested for a relationship between the amplitude changes during—compared to those after tsDCS. To take into account the possible differences in tsDCS modulatory response for BDNF met allele carriers (Lamy and Boakye, 2013), we assessed BDNF genotype in all subjects.

## Materials and methods

### Subjects

We included 10 healthy volunteers with a mean age of 23 (range: 20–29) years. All participants gave their written informed consent before data collection and the study protocol was approved by the local ethics committee of Twente (Enschede, The Netherlands).

### tsDCS

As announced, tsDCS was applied in two different electrode placement configurations (Figure 1). The amplitude was 2.5 mA and the duration 15 min using a NeuroConn DC-Stimulator PLUS (neuroCare Group GmbH, Munich, Germany). The electrode configurations chosen were: LSC-S: Lumbar Spinal Cord (T11)-left posterior Shoulder (Figure 1A) and ED: Equal Distance 7 cm above and below T11 (Lumbar Spinal Cord) (Figure 1B). LSC-S was applied in anodal configuration only, whereas for configuration ED the effect of both polarities was investigated. We refer to the polarity of the lower electrode for configuration naming for all configurations (e.g., ED-A and ED-C). Vertebra T11 was determined via manual palpation of the spinal processes, staring at vertebra C7 and counting until vertebra T11 was reached. This process was repeated three times, with the final position estimate determined by taking the mean of the three initial estimates. Sham stimulation, included in the utilized stimulation device, was achieved by applying a 110 μA pulse with a pulse-width of 3 ms and an interval of 550 ms for a duration of 15 min.

### H-reflex measurement

**Figure 2:**
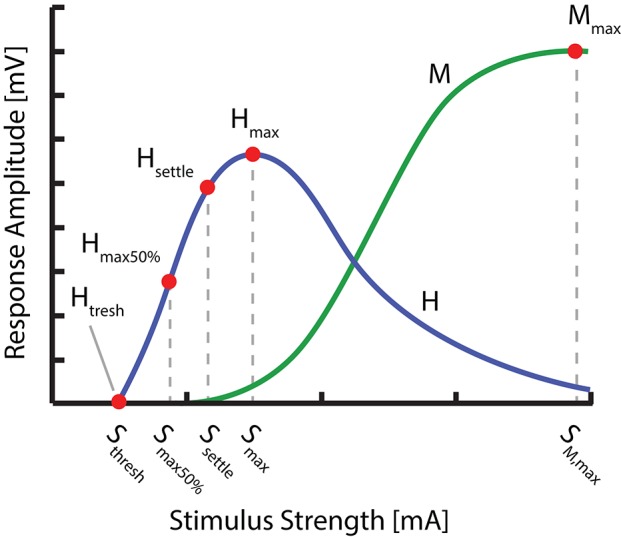
Overview of the distinct points measured within the H- and M- Wave recruitment curves: H-Reflex threshold (*H*_*thresh*_), 50% of H-reflex maximum (*H*_*max*50%_), the point at which the ascending part of the recruitment curve begins to settle (*H*_*settle*_), the maximum H wave (*H*_*max*_) as well as the maximum M-wave (*M*_*max*_).

To determine the changes induced to the H-reflex by tsDCS in one of the three configurations, we chose to characterize the H-reflex recruitment curve at four characteristic points (Figure 2): H-Reflex threshold (*H*_*thresh*_), 50% of H-reflex maximum (*H*_*max*50%_), the point at which the ascending part of the recruitment curve begins to settle (*H*_*settle*_) as well as the maximum H-wave (*H*_*max*_). These points were chosen for their ability: (1) to reflect the anticipated changes of the H-reflex recruitment curve (left/right shift, based on Lamy et al., 2012), or overall amplitude modulation, as well as (2) to sufficiently approximate the ascending part of the recruitment curve. The points were determined from a detailed H-reflex recruitment curve, recorded at the start of each experiment. The recruitment curve was sampled at stimulation intervals *I* (see: Experimental Protocol). Thereby, *M*_*max*_, *H*_*max*_, and *H*_*thresh*_ were determined manually, with *H*_*max*_ defined as the peak value of the average H-wave recruitment curve and *M*_*max*_ the amplitude immediately after peak settling amplitude of the M-wave recruitment curve. *H*_*thresh*_ was defined as the first visible H-Wave, in response to a stimulus. *H*_*settle*_ and *H*_*max*50%_ were determined via fitting of a sigmoid function *f*(*s*) to the recorded recruitment curve: $f\left(s\right)=H_{max}/\left(1+e^{-m\left(s-S_{max50\%}\right)}\right)$. Thereby, *m* is the function slope at *f*(*S*_*max*50%_), *S*_*max*50%_ the stimulus needed to evoke 50% of *H*_*max*_, *H*_*max*_ the maximum value of the recruitment curve and *S*_*max*_ the corresponding stimulation amplitude. *H*_*max*50%_ and *H*_*settle*_ are then defined as *f*(*S*_*max*50%_) and *f*(*S*_*settle*_) respectively, given $f^{\prime \prime }\left(S_{settle}\right)=\min\left(f^{\prime \prime }\left(s\right)\right)$. For each point, the closest multiple of *I* was chosen as a stimulation amplitude.

### EMG

Bipolar, EMG was recorded using a TMSi Porti amplifier (TMSi, Oldenzaal, NL) from the belly of the right lateral soleus muscle with electrode centers placed ~3 cm apart, 4 cm below the initiation of the gastrocnemius tendon. The sampling frequency was set to 2048 samples/s.

### Nerve stimulation

H-Reflex responses were evoked using electrical stimulation of the tibial nerve (Micromed Matrix Light, Micromed S.p.A., Mogliano Veneto, Italy). Adhesive active-cathodal (1.5 × 1.5 cm) and return -anodal (5 × 5 cm) electrodes were placed over the tibial nerve in the popliteal fossa and above the patella respectively. The stimulation consisted of a biphasic pulse with a pulse width of 0.5 ms and stimulation amplitudes ranging from 0 to 80 mA.

### BDNF genotyping

Saliva samples were collected (Oragene Dx, DNA Genotek Inc., Ottawa, Canada) from each subject. Subsequently all samples were analyzed to detect the BDNF Val66Met polymorphism using Taqman (rs6265). Additionally, BDNF concentration and sample purity (260/280) were detected.

### Experimental protocol

The experiment was set up in a randomized double-blind placebo controlled design, whereby both experimenter and subject were blinded with respect to the intervention type (real or sham). Interventions consisted of the three stimulation configurations and one sham stimulation. For each intervention, an individual experiment was performed in a randomized order with experiments planned with an interval of at least 7 days. The configuration by which sham was performed was randomized across subjects.

Subjects were instructed to avoid drinking coffee or consume other stimulants on the day of the experiment. Preparatory steps before attachment of EMG and tibial nerve stimulation electrodes included skin disinfection with alcohol, shaving and exfoliating of the desired skin section. With the subject lying on a medical bench in a prone position, EMG electrodes and nerve stimulation counter electrode in place, a handheld stimulation probe was used to determine the optimal position to stimulate the tibial nerve. Indicators for an appropriate stimulation position were a clear EMG response and visible contraction of the soleus, while excluding the contraction of other muscles such as the tibialis anterior, to avoid stimulation of the peroneal nerve. Additionally, an approximate H-reflex threshold was determined during this procedure, used for the determination of the needed stimulation increment for recruitment curve sampling.

**Figure 3:**
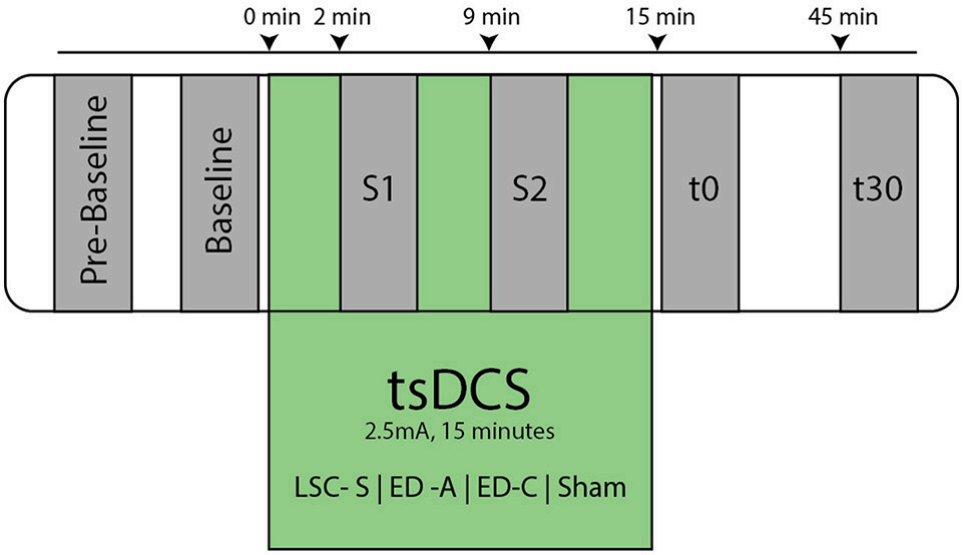
Overview of experimental protocol. At Pre-Baseline an initial recruitment curve mapping took place to determine characteristic points within the H- and M- Wave recruitment curves (see Figure 2). After an additional baseline measurement, the tsDCS intervention was started with an amplitude of 2.5 mA and a duration of 15 min. During tsDCS, the H-reflex is probed after 2 min (S1) and 9 min (S2), measuring only *H*_*settle*_, *H*_*max*_, and *M*_*max*_. Post tsDCS measurements follow immediately (t0) and 30 min (t30) after the intervention.

After placement of the active stimulation amplitude, the subject was comfortably seated in an inclined medical chair, head and arms supported (Ankle angle: ~110°, Knee angle: ~150°, Hip angle: ~120°, similar to Lamy et al., 2012). Thereafter, the protocol was executed as shown in Figure 3, for which the subject was instructed to remain entirely still and to avoid movement or muscle tension throughout the course of the experiment.

As a first step, an entire recruitment curve was measured at small intervals, later used to determine the stimulation amplitude of four relevant H-wave points (Figure 2). Starting at a stimulation amplitude at which no response was visible, the amplitude was increased gradually in predetermined intervals *I*, while measuring six times at each increment. *I* was set according to the previously approximated threshold amplitude. For thresholds below 10 mA, increments were set to threshold/10, otherwise an interval of 1 mA was used. The recruitment curve was sampled until reaching its declining portion after *H*_*max*_, after which the amplitude was increased at larger increments, until after the maximum M-wave was reached.

After completion of the initial curve mapping process, the stimulation amplitudes for *H*_*thresh*_, *H*_*max*50%_, *H*_*settle*_, *H*_*max*_, and *M*_*max*_ were identified within the recorded recruitment curve (see: H-reflex measurement:). These stimulation amplitudes were then held constant throughout the experiment.

After an additional baseline measurement, the tsDCS intervention was started. Post measurements were performed immediately after (t0) and 30 min following the intervention (t30). To assess the acute stimulation effects, additional measurements, 2 min (S1) and 9 min (S2) in the course of the intervention, were conducted. To reduce interference with effects of tsDCS, only *H*_*max*50%_, *H*_*settle*_, and *M*_*max*_ were measured at S1 and S2. The protocol was repeated for each electrode configuration and a sham condition.

### Data analysis

Data processing was performed with a custom Matlab script (Matlab v.2015a, MathWorks Inc., Natick, USA). EMG signals were high pass filtered at 5 Hz after which H- and M- wave peak-to-peak amplitudes were determined automatically. Thereafter, all amplitudes were normalized with their corresponding *M*_*max*_. Extreme outliers, such as null responses, were removed manually.

We expressed each obtained data point by its difference to baseline. This difference is normalized by the value of *H*_*max*_ at baseline and therefore expressed as a fraction of initial, overall H-reflex amplitude allowing comparison between sessions.

As an additional outcome-measure, we calculated the area below the sampled characteristic points, which gives an indication about the curve as a whole. Again, the area was expressed as its difference to the area calculated for its respective baseline. The resulting area difference was normalized by the overall area at baseline.

Because the H-reflex during stimulation was only measured at two sample points (*H*_*max*50%_ and *H*_*settle*_), we also calculated the area difference to baseline restricted to the interval between *H*_*max*50%_ and *H*_*settle*_. This was done in order to compare measurements during, to those before and after tsDCS.

### Statistics

Statistical analysis was performed using IBM SPSS Statistics v.23 (IBM Corporation, New York, USA). For each stimulation condition a Friedman's one-way ANOVA was used to test for significant effects in time and for each time interval for significant effects of stimulation configuration. This was performed on changes in curve area and data point amplitudes at *H*_*max*_ and *H*_*max*50%_. The significance level was set to *p* = 0.05. *Post-hoc* pairwise comparison was performed using Dunn's–test (Dunn, 1964) with an adjusted *p*-value of 0.0083. Effect size *r* was calculated via $r=\frac{Z}{\sqrt{2N}}$ with *N* being the number (10) of subjects (Pallant, 2013). Furthermore, we calculate the mean (*m*_*abs*_) and interquartile range (*IQR*_*abs*_) of the absolute difference from baseline for conditions found to be significant by *post-hoc* comparison.

To differentiate between a recruitment curve threshold shift and overall amplitude shift, we use Friedman repeated measures ANOVA to compare within measurement values for *H*_*max*50%_ and *H*_*max*_ for *post-hoc* measurements significant with respect to baseline. We thereby assume that a change in recruitment curve threshold, which is visible in a left or right shift of the recruitment curve, will result in a significantly larger amplitude change measured at *S*_*max*50%_ compared to those at *S*_*max*_. Consequently, an overall amplitude decrease will result in no significant difference between the changes at *H*_*max*50%_ and *H*_*max*_.

Furthermore, correlation analysis is used to rule out that changes in H wave could be attributed to a change in *M*_*max*_. In order to investigate differences between genotype groups, a Kruskal-Wallis test is performed for each measurement in which a significant difference was found, with the genotype as between group factor. For conditions across which a significant difference was found, we assess two-tailed Pearson and Spearman correlations by using the area between the corresponding measurement and baseline in a range from *H*_*max*50%_ to *H*_*settle*_.

## Results

### Subject safety

Throughout the course of the study, all subjects underwent the experiments without adverse effects, neither during or after the applied tsDCS, nor during the tibial nerve stimulation.

### Changes in H-reflex

**Figure 4:**
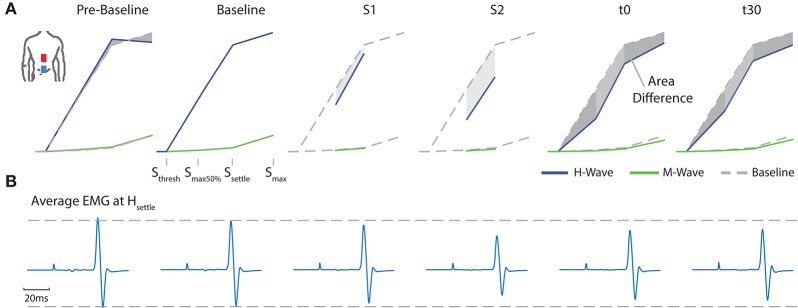
For an exemplary subject **(A)** all average H- and M- Wave datapoints are shown. For clarity, the figure exemplifies the area between a corresponding measurement and baseline. **(B)** Average EMG traces at *H*_*settle*_ for configuration ED-C.

**Figure 5:**
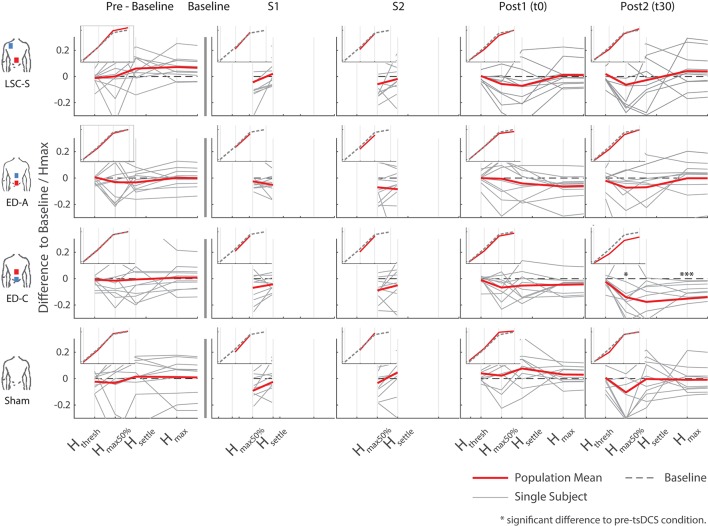
Overview of all differences to baseline, as a percentage of the corresponding *H*_*max*_ at baseline, for all configurations and measurements. The inlays show the average trend over all subjects with respect to a generic s-function representing baseline. Significant differences with respect to pre-intervention measurements arose for configuration ED-C, 30 min after intervention (last column).

**Figure 6:**
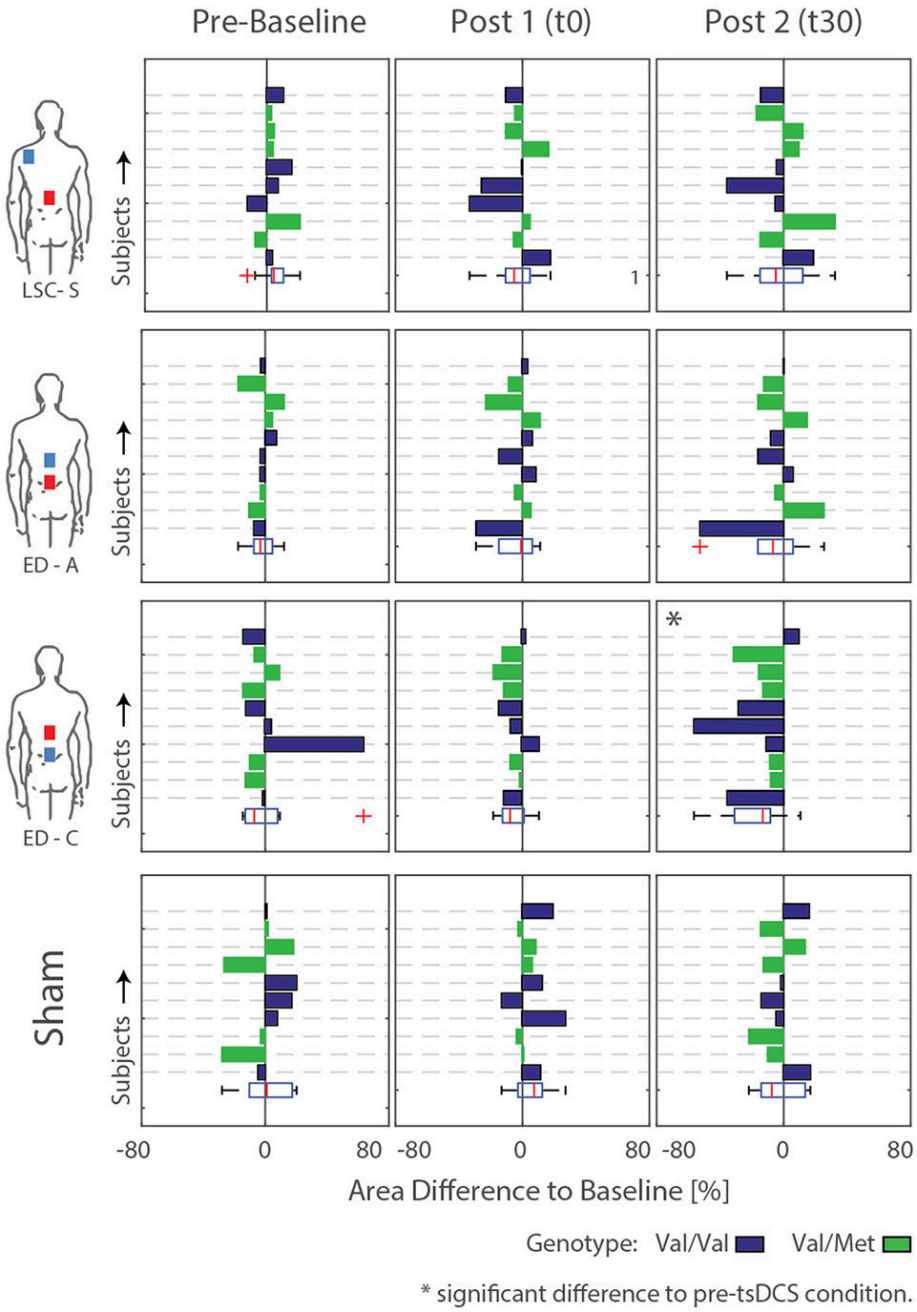
Difference areas between baseline and pre-baseline as well as post tsDCS (t0 and t30) for all subjects. Rows/Bars within each graph correspond to the measured subjects. Additionally, the subject genotype is color coded: Val/Val = Blue, Val/Met = Green. Significant differences to pre-tsDCS measurements are detected for configuration ED-C at time t30.

A typical measurement for an exemplary subject, including sampled H- and M-wave datapoints and average EMG traces at *s*_*max*50%_, for condition ED-C is illustrated in Figure 4. An overview of all H-Reflex datapoints, expressed as a percentage difference of *H*_*max*_ at baseline, for all subjects and conditions is shown in Figure 5. The total area, as a percentage difference of the area at baseline (see also Figure 4), quantifies the overall change (see Figure 6), whereas differences in mean datapoint amplitudes for *H*_*max*_ and *H*_*max*50%_ (Figure 5) can be interpreted as changes in overall amplitude and threshold respectively.

Responses to the different stimulation paradigms show high inter and intra subject variability. The changes observed for all sham measurements and those obtained during curve mapping before baseline give an indication of the natural changes that can be expected without intervention. Thereby the fluctuation in changes in area for all combined pre-baseline and sham measurements normalized by their baseline had an interquartile range of 21%. Similarly, for *H*_*max*_: IQR = 13%, and *H*_*max*50%_: IQR = 45%. Across subjects, most post-intervention changes are within the amplitude range of effects observed without intervention, as visible in Figures 5, 6.

For configurations LSC-S and ED-A, observed changes lie within the same standard deviation range as the sham condition. Furthermore, for both stimulation conditions, responses in either direction are visible, resulting in no statistically significant net-change with respect to baseline (Table 1). Similarly, for the sham condition, responses in both directions resulted in no statistically significant changes with respect to baseline.

**Table 1:** Statistical analysis of general differences within conditions and measurement times, across conditions, after Friedman repeated measures analysis.

	**Significance**		
	**Area difference**	***H*_*max*_**	***H*_*max*50%_**
**CONFIGURATION**			
LSC-S	0.51	0.16	0.51
ED-A	0.66	0.24	0.073
ED-C	0.033^*^	0.001^***^	0.033^*^
Sham	0.26	0.54	0.15
**MEASUREMENT TIME**			
Pre-Baseline	0.16	0.39	0.78
t0	0.06	0.05^*^	0.52
t30	0.095	0.06	0.78

*Bold highlights significance*.

* *p ≤ 0.05*,

*** *p ≤ 0.001*.

For configuration ED-C, differences in curve area (Figure 6) as well as data points *H*_*max*50%_ and *H*_*max*_ (Figure 5) reveal clear tsDCS effects post-intervention compared to baseline. This is indicated by overall significant changes in area [χ2_(3)_ = 8.76, *p* = 0.033], *H*_*max*_ [χ2_(3)_ = 16.56, *p* = 0.001], and *H*_*max*50%_ [χ2_(3)_ = 8.76, *p* = 0.033]. *Post-hoc* analysis reveals a significant difference of t30 to pre-baseline (*H*_*max*_: Z = 1.8, *p* = 0.011, *r* = 0.4, *m*_*abs*_ = 0.15, *IQR*_*abs*_ = 0.19) and baseline (area: Z = 1.7, *p* = 0.019, *r* = 0.38, *m*_*abs*_ = 0.22, *IQR*_*abs*_ = 0.24; *H*_*max*_: Z = 2.2, *p* < 0.001, *r* = 0.49, *m*_*abs*_ = 0.15, *IQR*_*abs*_ = 0.19; *H*_*max*50%_: Z = 1.7, *p* = 0.019, *r* = 0.38, *m*_*abs*_ = 0.4, *IQR*_*abs*_ = 0.24) measurements. To constrain the character of the observed recruitment curve changes, we compared the data points at *H*_*max*50%_ with those at *H*_*max*_, within configuration ED-C at time t30. Thereby no significant differences were found (*p* = 0.114). Thus, the population trend with respect to baseline for condition ED-C appears to be an overall H-reflex decrease instead of a curve shift to the right, revealed by a significant decrease in area, *H*_*max*50%_ and *H*_*max*_.

The observed effects in *H*_*max*50%_, *H*_*max*_ and curve area could not be explained by changes in nervous excitation during tibial nerve stimulation as changes in these measures were unrelated to changes in *M*_*max*_. We also explored whether changes during tsDCS (measurements S1 and S2) were predictive for changes post intervention. However, from correlation analysis it appeared that this was not the case.

Testing for time effects across configurations, Friedman's test reveals differences in *H*_*max*_ for measurements at t0 [χ2_(3)_ = 7.8, *p* = 0.05]. However, whereas the highest difference was found between configuration ED-A and sham (Z = −1.4, *p* = 0.09), *post-hoc* pairwise comparisons did not lead to significant results. Furthermore, analysis reveals a notable statistical trend [χ2_(3)_ = 7.56, *p* = 0.06] across conditions at time t30.

To explain the high variability across subjects, BDNF genotype was tested. Thereby, 5 out of 10 subjects were tested positive for the Vall66Met polymorphism. The corresponding subjects' genotype is included by coloring the bars in Figure 6. Generally, there is no consistent difference in stimulation response between genotype groups. For conditions exhibiting significant differences compared to baseline, Kruskal-Wallis' test reveals no significant differences between the responses exhibited by the two genotype groups. Thus, the high intersubject variability, which remains within genotype groups, cannot be explained by or, otherwise stated, prohibits explanation of an effect of subject genotype.

## Discussion

The goal of this study was to investigate electrode placement specific changes of lumbar trans-spinal direct current stimulation on the soleus H-reflex before, during and after intervention. We introduced a new electrode placement configuration (ED), which generates an electric field vector dominant in longitudinal direction at lumbar spinal motoneuron level, by placing both electrodes equidistant above and below the lumbar spinal cord (Kuck et al., 2017). We show that the newly introduced electrode configuration (ED-C) was able to induce significant changes to the approximated H-reflex recruitment curve 30 min after intervention. This was indicated by a significant and consistent depression of *H*_*max*_, *H*_*max*50%_, and area. The equal distance placement in anodal setting (ED-A) had no significant effect, which confirms the polarity dependency often observed in DC stimulation protocols. Additionally, the effects observed post tsDCS were unrelated to the deviations from baseline measured during DC stimulation. Strikingly, we were not able to observe the effects previously reported for configuration LSC-S (Lumbar Spinal Cord—Shoulder), which had been shown to induce a significant left shift of the H-reflex recruitment curve (Lamy et al., 2012).

The specific post intervention response to stimulation with configuration ED-C appears to be an overall amplitude reduction of the H-reflex, indicated by a relative decrease in *H*_*max*_, *H*_*max*50%_ and area. This is qualitatively different from the left shift reported after anodal tsDCS (LSC-S) in previous studies and may consequently indicate the involvement of different working mechanisms. Cellular targets of lumbar spinal DCS that have been suggested by previous studies, are the Ia-motoneuron synapse (Lamy et al., 2012; Hubli et al., 2013; Kuck et al., 2017), Ia-presynaptic inhibition (Yamaguchi et al., 2013), or channels mediating persistent inward current excitability (Elbasiouny and Mushahwar, 2007).

We argue, that conductivity changes at the Ia-motoneuron synapse seem less likely, as this would lead to a left shift or right shift of the H-Reflex recruitment curve, not an amplitude modulation (Kuck et al., 2017). Thus, also changes in Ia-presynaptic inhibition appear unlikely. It can however not be excluded, that the observed effect in H-Reflex reduction could in part be caused by a downregulation of Ca^2+^ persistent inward current. Elbasiouny and Mushahwar investigated the effect of motoneuron polarization on spinal motoneuron firing and PIC modulation (Elbasiouny and Mushahwar, 2007). Thereby a constant EF was able to directly surpress motoneuron firing by reducing Ca^2+^ current. In analogy to that, the tsDCS generated electric field in this study, could have led to a similar polarization profile of the lumbar spinal motoneurons (Kuck et al., 2017), which may in turn have resulted in a downregulation of motoneuron activity.

In an effort to understand inter-individual response differences, we investigated the relationship between tsDCS acute and after effects as well as differences in BDNF genotype. To test the relationship between acute and after effects, we correlated the conditions found to be significant, to the changes from baseline measured during tsDCS within the same experiment. However, no relationship was detected.

A possible reason that a relation between acute and long-term effects was not found in this study, could be that the stimulation intensities usually used for human subjects were too low to elicit measurable acute effects. This is different in animal and in *in-vitro* DC experiments, which show measurable acute effects during DC stimulation, which scale with stimulation intensity (Ahmed, 2011; Rahman et al., 2013). The relationship between DCS acute and after effects is complex, as shown by Ahmed (2011), whereby MEP evoked muscle twitches in the hindlimb where inversed during, compared to after tsDCS. Furthermore, this directional relationship was altered by associative stimuli during tsDCS.

Genetic dependencies for the response to tsDCS have been shown for Met allele carriers of brain derived neurotropic factor (BDNF) (Val66Met polymorphism) (Lamy and Boakye, 2013; Wiegand et al., 2016). However, BDNF genotyping in our subject population reveals that the level of variability remains within the two genotype groups and thus no statistical difference between the responses exhibited by the two subject groups was found. This does however not rule out the influence of other genetic dependencies (Wiegand et al., 2016).

We did not observe a consistent recruitment curve left shift after tsDCS with configuration LSC-S, as reported previously (Lamy et al., 2012). Based on Lamy et al. we had expected a substantial increase of *H*_*max*50%_ with respect to baseline (for configuration LSC-S at time T30). However, with a mean difference from baseline for *H*_*max*50%_ of −6.3% (95% CI [−20.81, 8.26%]), the population response observed here is substantially different from that. However, this is in line with observations by Hubli et al. who showed no significant modulatory effects after anodal tsDCS tested in healthy individuals (Hubli et al., 2013). We therefore assume that the absence of a modulatory effect after tsDCS in configuration LSC-S, must be attributed to experimental and/or subject-specific factors.

The two main methodological differences between the protocol used here and the one of Lamy et al. are the amount of measurements taken during tsDCS as well as sample size. For the former, Lamy et al. sampled two complete H-reflex recruitment curves during tsDCS at a stimulus frequency of 0.33 Hz, and a measurement time of 3–4 min for each curve. In contrast to that, we intentionally reduced the number of measurements during tsDCS, to prevent interactions with the artificially induced neural activity and therefore influence intervention outcome. With each measurement lasting ~1–2 min (thus overall 2–4 min during tsDCS), the amount of induced neural activity was substantially lower as compared to the protocol performed by Lamy et al. (6–8 min during tsDCS). Along this line, Hubli and colleagues did not measure during tsDCS and stimulation was applied during rest, thus reducing neural activity during DC stimulation to a resting level (Hubli et al., 2013). Since the outcome of DC stimulation is thought to be neural activity dependent (Bikson et al., 2013), the agreement of our results with those reported by Hubli et al. (2013) and the discrepancies with those observed by Lamy et al. may be explained via the differences in induced neural activity during tsDCS.

With regards to sample size, we included a smaller number of subjects (N = 10) compared to Lamy et al. (N = 17), which may suggest limited statistical power to show an otherwise significant effect. However, based on the mentioned population mean for *H*_*max*50%_, 30 min after tsDCS with configuration LSC-S, the responses obtained here are substantially different from those reported by Lamy et al. Furthermore, our results agree with those of Hubli et al. who had included the same number of subjects (N = 17) as Lamy et al. Out of these reasons, it is unlikely that sample size is able to account for the mentioned differences in intervention outcome.

## Conclusion

The presented results are a further step toward forming a basic understanding of tsDCS and may potentially contribute to a more targeted application in the future. In the light of the knowledge obtained by others, the overall reduction of the H-reflex after stimulation with configuration ED-C indicates that by changing EF direction with respect to the target structure the network response can be changed. This implies that different cellular targets may be dominant depending on EF orientation, which is in line with current state of the art knowledge. Against our expectations, we were not able to observe the same recruitment-curve left shift for configuration LSC-S as previously reported by others, which could be accounted for by methodological or subject specific differences as discussed. In addition to the depression effects discussed earlier, this highlights the complexity of the underlying mechanisms, which have to be understood before tsDCS can find its way into clinical application.

## Author contributions

AK, DS, HvdK, and EvA took part in initial planning. AK carried out the experiments and data analysis. AK, DS, HvdK, and EvA contributed to the interpretation of the results. AK took the lead in writing the manuscript. All authors provided critical feedback and helped shape the research, analysis, and manuscript.

### Conflict of interest statement

The authors declare that the research was conducted in the absence of any commercial or financial relationships that could be construed as a potential conflict of interest.
